# A Practical Source of Inulin

**Published:** 1913-02

**Authors:** 


A Practical Source of Inulin.—Editorial in *Western Med. Rev.,* November, 1912. Referring to Strauss's articles in the *Deutsche Med. Woch.,* March 7, and *Berliner Klin. Woch.,* June 24, 1912, the editor makes some very caustic criticisms as to indefinite descriptions of plants and plant parts by Strauss (which may, however, have been perfectly clear to a German audience) and emphasizes the practical fact that inuloid, isomeric with inulin, is abundant in the tubers of the ordinary Jerusalem artichoke, which are so cheap as to be used for cattle food. Inulin from elecampane is expensive. He thinks that viper's grass (black salsify or scorsonera hispanica), the German plant recommended by Strauss, furnishes larger and more tasty roots than the ordinary salsify (vegetable oyster) and that it should be cultivated in the United States.

